# Exercise interventions for post-stroke depression: A protocol for systematic review and meta-analysis

**DOI:** 10.1097/MD.0000000000024945

**Published:** 2021-02-26

**Authors:** Wei Zhang, Yi Liu, Jing Yu, Qin Zhang, Xiaoyan Wang, Yongqing Zhang, Yongli Gao, Lei Ye

**Affiliations:** Department of Emergency Medicine, West China Hospital, School of Nursing, and Disaster Medical Center, Sichuan University, Chengdu, China.

**Keywords:** exercise, physical activity, Post-stroke depression, prognosis

## Abstract

**Background:**

Post-stroke depression (PSD) is one of the most common neuropsychiatric complications after stroke and is associated with increased risk of death and poor functional outcomes. Strong evidence shows that exercise has benefits for depression. However, it is not clear whether exercise has benefits specifically for PSD. This study aims to explore the effects of exercise on PSD and to establish safe and effective exercise prescriptions.

**Methods and analysis:**

The PubMed, Cochrane Library and EMBASE, databases will be searched using prespecified search strategies. Randomized controlled trials and non-randomized prospective controlled cohort studies regarding exercise for PSD will be included. The primary outcomes are depression scale and stroke outcome. The secondary outcomes are the occurrence of adverse events, cognitive function, quality of life indices, and the expression of nerve cell factors. The methodological quality of each study will be evaluated by the physiotherapy evidence database scale. The heterogeneity will be evaluated using the *I*^2^ test. If *I*^2^ > 50%, random effects models will be used in the analysis; otherwise, fixed effects models will be used to pool the data.

**Results:**

This study will assess the efficacy and safety of exercise for PSD.

**Conclusions:**

Our findings will be helpful for clinicians to re-examine the clinical decision-making in the treatment of PSD, by assessing the efficacy of a promising treatment modality for patients with PSD.

**Ethics and dissemination:**

Ethical approval is not required because this study is a secondary analysis. The results of this study will be disseminated through journals and academic exchanges.

**Systematic review registration number:**

INPLASY202110100.

## 1 Introduction

Stroke is the second leading cause of death and disability after ischemic heart disease and the third-leading cause of disability-adjusted life-years.^[1]^ In the United States, approximately 795,000 people have a stroke every year. On average, there is a stroke every 40 seconds and a stroke-related death every four minutes.^[2]^ The cost of informal care for stroke patients accounts for more than half of the total cost of informal care for cardiovascular disease.^[3]^ Stroke places a huge burden on affected individuals and health systems, and can lead to functional limitations and psychological disorders. Much attention is paid to motor disorders and physical disabilities following stroke, but the associated psychological disorders are often ignored. However, stroke has been associated with several psychological disorders, including anxiety, apathy, depression, cognitive impairment, mania, and mental illness.^[4–6]^ Post-stroke depression (PSD) is one of the most common neuropsychiatric complications after stroke. Approximately 31% of stroke patients develop depression at some point within five years after the stroke.^[7–9]^ The clinical manifestations are physical dysfunction, depression, slow thinking, and a series of autonomic nervous dysfunctions. Serious PSD reduces the patient's confidence in therapy and recovery, thus affecting the functional rehabilitation process, deteriorating the patient condition, and increasing mortality.^[10]^

Drug and psychological therapy have been traditionally used to treat PSD. In several published reviews, drug therapy and psychological therapy were shown to be effective for depression in the elderly and in people with associated physical illness.^[11–13]^ However, because PSD may differ in important ways, it is not appropriate to extrapolate such data to PSD. The use of antidepressants can counteract the negative effects of depression on functional recovery after stroke.^[14]^ However, it will also lead to bleeding, cardiovascular side effects, metabolic enzyme inhibition, effects on the central nervous system, and other adverse events.^[15,16]^ Psychotherapy is commonly applied to treat the psychological problems of patients with PSD, and the available evidence suggests that pharmacological interventions and psychological therapy may prevent depression and improve mood after stroke. However, these conclusions are characterized by very low levels of evidence.^[17]^

Some studies have shown that exercise may be a complementary treatment for depression.^[18–21]^ Exercise may affect depressive symptoms through various mechanisms. For example, the hypothalamus-pituitary-adrenal axis and immune function may be maladjusted in depression, resulting in elevated cortisol levels. Exercise can improve the regulation of the hypothalamus-pituitary-adrenal response and increase immunity.^[22–24]^ A meta-analysis of adults with depression (not including PSD) showed that exercise plays a positive role in people with non-stroke depression.^[25]^ However, a previous meta-analysis of stroke patients with depression indicated that only higher intensity exercise had a significant effect on depressive symptoms, while lower intensity exercise protocols did not.^[26]^ The use of antidepressant medications was not documented in most studies, so that its potential confounding interaction with exercise could not be assessed. After that systematic review, several well-designed trials have been conducted to investigate the association between exercise and risk of PSD, with conflicting conclusions. Moreover, there is a lack of consensus on the timing, type, intensity, and duration of exercise in patients with PSD. Therefore, it is necessary to conduct a new systematic review and meta-analysis to evaluate whether exercise is effective for PSD.

## 2 Methods

This study follows the reporting guidelines of the Preferred Reporting Items for Systematic Reviews and Meta-Analysis for Protocols 2015. The research method is described according to Cochrane Handbook for Systematic Reviews of Diagnostic Test Accuracy. This protocol was registered on the International Platform of Registered Systematic Review and Meta-analysis Protocols (INPLASY202110100).

### 2.1 Eligibility criteria

Studies that meet all of the following criteria will be included in the meta-analysis:

#### 2.1.1 Types of studies

Only randomized controlled trials and non-randomized prospective controlled cohort studies of early post-stroke exercise rehabilitation training will be considered. Other types of research, such as observational studies, animal trials, research programs, and ongoing trials, will be excluded.

#### 2.1.2 Types of participants

Studies enrolling patients diagnosed with PSD with sufficient medical stability at the beginning of the intervention will be considered. No limits will be placed on age, race, sex, or type, and severity of stroke. The diagnosis of stroke should be based on computed tomography magnetic resonance imaging, or clinical criteria. Meanwhile, the diagnosis of depression should follow the guidelines of the International Classification of Diseases, Tenth Edition, or the Diagnosis and Statistical Manual of Mental Disorders. Patients should have been diagnosed with both stroke and depression to be considered. The study will not include patients with other diseases seriously disrupting the physical condition of the whole body, or who had been previously diagnosed with a mental disorder.

#### 2.1.3 Types of interventions

The study will include any exercise intervention, without limits on exercise type (such as aerobic exercise, resistance training, combined aerobic and anaerobic training, upper or lower limb training, Tai Chi and yoga), frequency, intensity, or duration. Interventions in any environment will also be included, including outpatient rehabilitation, family rehabilitation, community rehabilitation, and hospital rehabilitation. Moreover, the control group should not have received any exercise intervention.

### 2.2 Types of outcome measures

The main outcomes are depression scale and stroke outcome. The depression scale can be assessed by the Beck Depression Inventory (BDI) or clinician-rated scales such as the Hamilton Depression Rating Scale. Stroke outcomes can be assessed by the improved Rankin scale (MRS). The MRS score ranges from 0 to 6, including 2 for mild disability and 6 for death.

Secondary outcomes will include the occurrence of adverse events, such as falls, pain, injury, death; any outcome related to cognitive function, including attention and processing speed, memory, language and global cognition, and executive function; quality of life indices, such as the World Health Organization Quality of Life; indices of life functions, such as the Activity of Daily Living scale and the expression of nerve cell factors (such as serotonin and IL-23). The Hamilton Depression Rating Scale scale consists of 21 questions. The score of the scale can be used to divide the subjects into one of the following groups: 0–7 normal; 7–13 mild depression; 14–18 moderate depression; 19–22 severe depression; >22 extremely severe depression. The degree of functional dependence on activities of daily living can be assessed using the Barthel Index (BI).

### 2.3 Search strategy

Two reviewers (YL and QZ) will independently search the PubMed, EMBASE, and Cochrane Library databases, without limits on retrieval time. The search keywords will be Medical Subject Headings (MeSH) terms and free text words, used separately or in combination. The main keywords will include: Cerebrovascular Apoplexy, Cerebrovascular Stroke, Cerebrovascular Accident, Acute Stroke, Acute Cerebrovascular Accident, Post-stroke Depression, Depression, Exercise, Exercise Therapy, Physical exertion. The keywords related to the interventions of interest will include the following: Cardiovascular Training, Cardiopulmonary Training, Aerobic Training, Endurance Training, Resistance Training, Cycling, Treadmill, Tai Chi, and Yoga. The selected articles must contain a detailed description of the exercise intervention, including type, frequency, duration, and intensity of the exercise. In addition, the references of the retrieved studies will be searched manually. All problems arising in the retrieval process will be discussed and solved by the two examiners and, if necessary, a third reviewer (WZ) will participate in the discussion and solution.

### 2.4 Data collection and analysis

#### 2.4.1 Selection of studies

**Figure 1:**
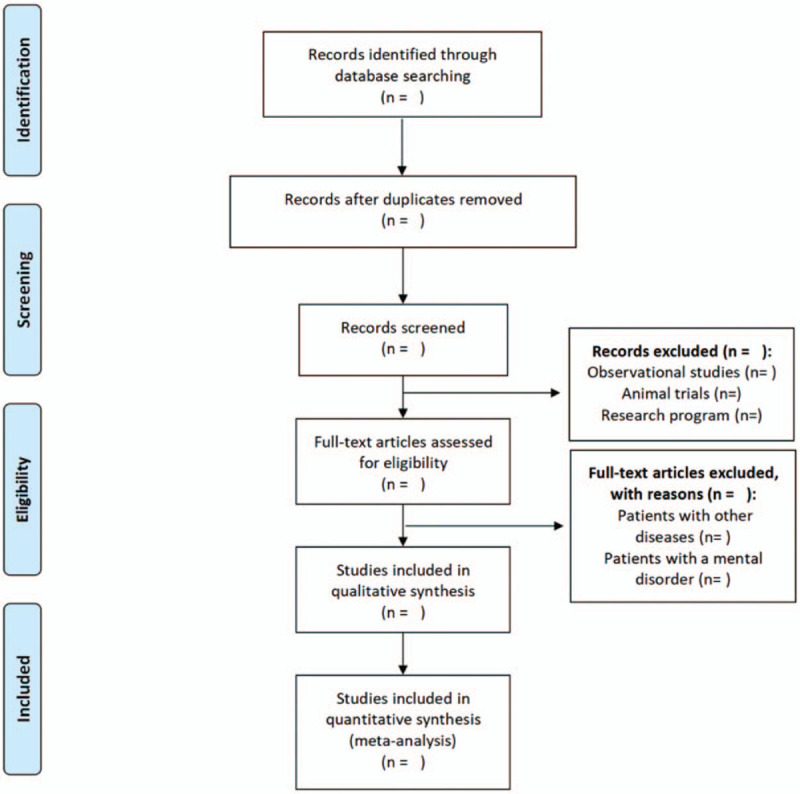
Flow diagram of this study selection.

All records will be imported into Endnote X9.3.3 (Clarivate Analytics, Philadelphia, Pennsylvania, USA) for filtering. Two independent reviewers (YL and QZ) will randomly sift through 20% of the search records, evaluate their titles or abstracts, and discuss their results until consensus is reached. If the consistency between the two researchers is high (greater than 80%), the remaining records will be filtered by a single reviewer (YL). If the consistency is less than 80%, all remaining records will be screened independently by two reviewers. We will retrieve the full text of all records that have been screened by title or abstract and are considered likely to meet the criteria. The reviewer will conduct a full-text evaluation of the studies that may meet the criteria but whose status is uncertain and collect the reasons for the exclusion. References for all eligible studies will also be screened to identify further eligible studies. All included documents will be read in full by the reviewer. During the screening process, if there are any unresolved differences, the third reviewer (WZ) will participate in the resolution. The research selection process will be presented in the form of a flowchart (Fig. 1).

#### 2.4.2 Data collection and management

The data will be extracted into a standardized Excel table. The fields will include the study ID (author and year), title, demographic characteristics (age, sex, race, time, location, type and severity of PSD), number of participants, study design, type of intervention (mode, intensity, frequency, duration of exercise), control group, place of intervention (hospital, community, family) and outcomes of interest (depression assessment scale, adverse events, results related to cognitive function, Activity of Daily Living, World Health Organization Quality of Life, and expression of nerve cell factors). The full text of the literature will be read again after data extraction to determine whether errata have been published or studies have been withdrawn. A reviewer (YL) will use two randomly selected studies to test the data extraction tables for user-friendliness and integrity. The two reviewers (YL and QZ) will then perform calibration links by independently and randomly extracting 30 per cent of the sample data included in the study. If the number of included studies is less than 15, three studies will be randomly selected for independent data extraction by two reviewers. If the data extraction consistency of the two reviewers is high (greater than 80%), the rest of the research data extraction will be performed by a single reviewer (YL). If the original consistency is less than 80%, the data will be extracted independently by the two researchers. Any differences arising during this phase will be discussed and resolved by the two reviewers, and by the third reviewer (WZ) if consensus cannot be reached. For documents with incomplete data or content, the reviewer will contact the author by e-mail, and the study will be excluded if the missing information cannot be obtained.

### 2.5 Statistical analysis

#### 2.5.1 Study quality assessment

We will use the recommended scoring, evaluation, development, and evaluation (rating) system to evaluate the quality of evidence. The scoring method specifies four quality levels: high, medium, low, and very low. The highest quality level corresponds to evidence from randomized trials. The reviewer can downgrade the evidence from a randomized trial to medium, low, or even very low depending on several factors.

#### 2.5.2 Risk of individual studies

Two independent reviewers (LYG and XYW) will assess the risk offset of all studies. The methodological quality of each study will be evaluated by the physiotherapy evidence database (PEDro) scale.^[27]^ The PEDro scale is used to evaluate the methodological quality of physiotherapy randomized controlled trials. The scale consists of 11 items, including 10 related to the internal validity and one to the external validity of clinical trials. The last item does not affect the internal or statistical validity of the trial and is not usually included in the calculation of the PEDro scale scores. In this study, only internal effects will be considered to assess risk offset. A PEDro total score ≥ 6 is considered high quality, 4 or 5 is considered medium quality, and <4 is considered low quality. We will exclude studies with a PEDro score of less than 4.

#### 2.5.3 Risk across studies

We will use funnel plots to evaluate publication bias. If there is no bias in the included studies, the points on the funnel plot will be symmetrically distributed around the estimated true values of the independent research effect points, showing an inverted symmetrical funnel shape. If there has been bias, the funnel plot will show asymmetry, and the more obvious the asymmetry, the greater the degree of bias.

#### 2.5.4 Data synthesis

The study will strictly follow the Preferred Reporting Items for Systematic Reviews and Meta-Analysis statement. We will use Stata V16.0 and Revman V5.3.5 for the calculation of mean deviation, standard deviation, confidence interval, and *P*-value. For continuous variables, we will use the standardized mean difference and its 95% confidence interval as the summary statistics of the meta-analysis. We will calculate the depression score before and after intervention in each group using the weighted average difference. For dichotomic data (such as the occurrence of adverse events), the Mantel-Haenszel method will be used to calculate the combined odds ratio. For variables that cannot be quantitatively analyzed, the results will be described in a narrative way. The total incidence of complications will be summarized by 95% confidence intervals. The heterogeneity between the included studies will be evaluated using the *I*^2^ test. If *I*^2^>50%, the studies will be considered to have high heterogeneity, and random effects models will be used in the analysis; otherwise, the data will be analyzed using fixed effects models.

#### 2.5.5 Subgroup analysis

If enough randomized controlled trials are selected for inclusion, we will conduct a subgroup analysis when there is significant heterogeneity in the trials. This will be conducted according to age, country, sex, intervention mode, and type of intervention between the experimental group and the control group. The intervention methods can be divided based on exercise type (aerobic exercise, anaerobic exercise, yoga, Tai Chi), frequency (< 3 times a week vs ≥ 3 times a week), duration (<12 weeks vs ≥ 12 weeks), and intensity (< 70% vs ≥70% heart rate reserve/VO2 peak).

#### 2.5.6 Sensitivity analysis

If necessary, we will also conduct a sensitivity analysis, by eliminating one by one the low-quality studies included and calculating the combined value of the remaining studies to observe whether the results change. If there are significant changes, the result will be considered to be unstable, otherwise it will be considered to be stable.

### 2.6 Patient and public involvement

No patient or public will be involved.

### 2.7 Ethics and dissemination

Because this study is a secondary analysis, it does not need to be ethically reviewed. The results of this study will be disseminated through peer-reviewed publications, journals, and academic exchanges.

## 3 Discussion

Through the proposed systematic review, we intend to assess the impact of exercise interventions on PSD. Exercise is a very common, convenient, non-invasive, and relatively inexpensive form of treatment, and strong evidence supports its negative association with depression. However, specific evidence about the effectiveness of exercise in PSD is still insufficient, which prevents it from being successfully used among the potential treatment options. Therefore, the need for this protocol is clear. To the best of our knowledge, it will be the first synthesis of the currently available publications to evaluate the efficacy and safety of exercise in patients with PSD. Given the narrow scope of our research questions, there may be only a few randomized controlled trials that can be included in the proposed systematic review, and the quality of the included studies will vary. Even if this happens, we still hope that the results will be useful as a summary of the existing evidence and provide some preliminary guidance for existing PSD practice and future research. This protocol aims to provide a framework of evidence on exercise therapy for patients with PSD that could be used by health care providers worldwide.

## Acknowledgment

This work was supported financially by grants from Sichuan Science and Technology Program (No. 2020YFS0154, 2020YFSY0014), 1•3•5 Project for Disciplines of Excellence-Clinical Research Incubation Project, Sichuan University West China Hospital (No. 2018HXFH001, 2018HXFH027, and 2020HXFH050), Sichuan University West China Nursing Discipline Development Special Fund Project (HXHL20046, HXHL19023), Chengdu Science and Technology Bureau (2019-YF05-00322-SN), National Clinical Research Center for Geriatrics, West China Hospital, Sichuan University (Z20191009)

## Author contributions

**Conceptualization:** Qin Zhang.

**Methodology:** Jing Yu.

**Project administration:** Xiaoyan Wang.

**Resources:** Yongli Gao, Lei Ye.

**Validation:** Yongqing Zhang.

**Writing – original draft:** Yi Liu.

**Writing – review & editing:** Wei Zhang.
